# First-line enfortumab vedotin-pembrolizumab versus nivolumab plus gemcitabine-cisplatin in metastatic urothelial cancer: a cost-effectiveness study

**DOI:** 10.3389/fpubh.2026.1723784

**Published:** 2026-02-12

**Authors:** Haojie Ying, Bin Fu

**Affiliations:** 1 The Fourth School of Clinical Medicine of Zhejiang Chinese Medical University, Hangzhou, Zhejiang, China; 2 Institute of Digital Traditional Chinese Medicine, Zhejiang Chinese Medical University, Hangzhou, Zhejiang, China

**Keywords:** cisplatin, cost-effectiveness, enfortumab vedotin, gemcitabine, metastatic urothelial carcinoma, nivolumab, pembrolizumab

## Abstract

**Introduction:**

Recent phase III programs, EV-302 and CheckMate-901, showed that enfortumab vedotin plus pembrolizumab (EV + P) and nivolumab with gemcitabine-cisplatin (N + GC) deliver superior clinical outcomes when used as initial therapy for advanced urothelial cancer. What remains uncertain is their relative economic value when assessed under Chinese and US payer conditions. To address this gap, we compared the value for money of EV + P versus N + GC as first-line management for la/mUC from the perspectives of healthcare payers in China and the US.

**Methods:**

We performed a model-based economic evaluation using a time-dependent state-transition framework implemented in TreeAge Pro (2022). Health benefits were measured as quality-adjusted life-years (QALYs) derived from health-state utilities, and comparative value was expressed as incremental cost-effectiveness ratios (ICER). The robustness of the model was assessed through one-way sensitivity analysis (OWSA) to evaluate the impact of key parameter uncertainties.

**Results:**

In the US, EV + P cost $1,863,624.32 and provided 3.34 QALYs, while N + GC cost $881,979.07 for 2.36 QALYs, resulting in an ICER of $1,001,626.19 per QALY, exceeding the $150,000/QALY threshold. In China, EV + P cost $485,374.69 and provided 2.95 QALYs, compared to $203,811.61 for 2.15 QALYs with N + GC, yielding an ICER of $351,960.68/QALY, above the $40,451.64/QALY threshold. Therefore, N + GC is the more cost-effective first-line strategy in both countries.

**Conclusion:**

Under current pricing and reimbursement assumptions, N + GC is economically preferable to EV + P as a first-line strategy for la/mUC in both the US and China. EV + P may warrant consideration only in tightly selected scenarios or with substantial coordinated price reductions and policy changes. Meanwhile, it should be noted that due to the inherent limitations of the indirect comparison method between drugs, the conclusions of this study should be regarded as exploratory analysis results.

## 1 Introduction

In 2022, bladder cancer ranked ninth worldwide for incidence and 13th for mortality. In China, an estimated 92,883 new cases placed bladder cancer 11th among all cancers, with a 5-year incidence estimated at 2.5 per 100,000. In the US, 80,404 new cases were recorded, sixth nationwide (1). Urothelial carcinoma, accounting for 90% of bladder tumors, is clinically burdensome and frequently aggressive (2, 3). Because many patients present at advanced stages, outcomes remain poor, reinforcing the need for more effective first-line options that extend survival.

Platinum-based chemotherapy remains the guideline-recommended first-line therapy for metastatic urothelial carcinoma, yet survival outcomes are limited (4). Owing to demonstrated benefit, immune checkpoint inhibitors have become integral to cancer care, including in urothelial carcinoma.

EV-302 (KEYNOTE-A39) is a phase 3, open-label, randomized, controlled trial evaluating enfortumab vedotin plus pembrolizumab (EV + P) versus platinum-based chemotherapy in previously untreated patients with locally advanced or metastatic urothelial carcinoma (la/mUC). The study demonstrated that the combination nearly doubled median overall survival (OS) and significantly prolonged median progression-free survival (PFS), with higher overall and complete response rates compared with chemotherapy. These benefits were observed across a broad patient population, with benefit observed regardless of PD-L1 status, ability to receive cisplatin, or baseline liver metastases (5). On the strength of these pivotal data, the 2025 CSCO Guidelines for Urothelial Carcinoma assigned first-line, Class I (Level 1A evidence) recommendation to EV + P, establishing it as a new standard initial-line therapy for advanced UC in China (6).

CheckMate-901 demonstrated that nivolumab plus gemcitabine-cisplatin (N + GC) confers significant first-line benefits in advanced UC, improving both OS and PFS versus chemotherapy by itself (7). *Post-hoc* analyses further indicated particularly pronounced efficacy in the lymph-node-only subgroup: median OS approached 4 years, and over 60% achieved complete tumor remission. Among cisplatin-eligible Chinese patients with unresectable/metastatic UC, N + GC showed a favorable benefit–risk profile (7), supporting its adoption as a new standard of care.

Both NCCN and NMPA have listed N + GC and EV + P as Category 1 initial-line options for UC (8–10). Although both regimens demonstrate superior efficacy to carboplatin plus gemcitabine, a number of recent pharmacoeconomic studies have found that neither regimen is more cost-effective than carboplatin-gemcitabine (11–14). To date, however, no economic evaluation has directly compared nivolumab + gemcitabine-cisplatin with EV + P. Such an analysis is particularly valuable for patients and decision-makers who prioritize clinical benefit while remaining mindful of costs. Accordingly, we conducted an economic evaluation of these two initial-line strategies for la/mUC from the healthcare payer perspectives in China and the US.

## 2 Patients and methods

### 2.1 Patient treatment

The clinical inputs for this economic evaluation were sourced from two first-line, phase III randomized trials in untreated, unresectable UC or mUC (Supplementary Table S1): EV-302 (enfortumab vedotin + pembrolizumab) and CheckMate-901 (nivolumab + gemcitabine-cisplatin). Our study included patients consistent with the EV-302 and CheckMate-901 eligibility criteria: adults with radiographically and histologically confirmed, unresectable la/mUC; RECIST v1.1-measurable lesions, ECOG PS 0–1, and no history of systemic therapy for advanced disease (7, 15).

In EV-302, enfortumab vedotin 1.25 mg/kg was infused on days 1 and 8, together with pembrolizumab 200 mg on day 1, within a 21-day cycle. Pembrolizumab could be given for ≤35 cycles, whereas enfortumab vedotin had no preset cap; therapy continued until disease progression or intolerable toxicity (protocol-defined stopping), whichever occurred first (15). In CheckMate-901, the experimental arm received nivolumab 360 mg IV on day 1 plus gemcitabine 1,000 mg/m^2^ on days 1 and 8 and cisplatin 70 mg/m^2^ on day 1, repeated every 21 days for up to six cycles, followed by maintenance nivolumab 480 mg IV every 4 weeks for as long as 2 years or until progression or unacceptable adverse effects (7).

Per NCCN and CSCO guidance (6, 10), we modeled second-line therapy as gemcitabine-cisplatin for cisplatin-eligible patients: gemcitabine 1,000 mg/m^2^ IV on days 1 and 8 plus cisplatin 70 mg/m^2^ IV on day 1. Third-line management after further progression was assumed to be best supportive care (BSC). For cost-effectiveness analysis, we used representative patient profiles to determine drug dose calculations and medical cost inputs: in China, weight 65 kg (13) and body surface area 1.72 m^2^ (16); in the US, weight 70 kg (13) and body surface area 1.86 m^2^ (17).

All clinical inputs (eligibility, dosing, maintenance rules, and subsequent-therapy allowances) were abstracted directly from the trial protocols and primary publications; because only published data were used, no new ethics approval was required.

### 2.2 Markov model review

**Figure 1:**
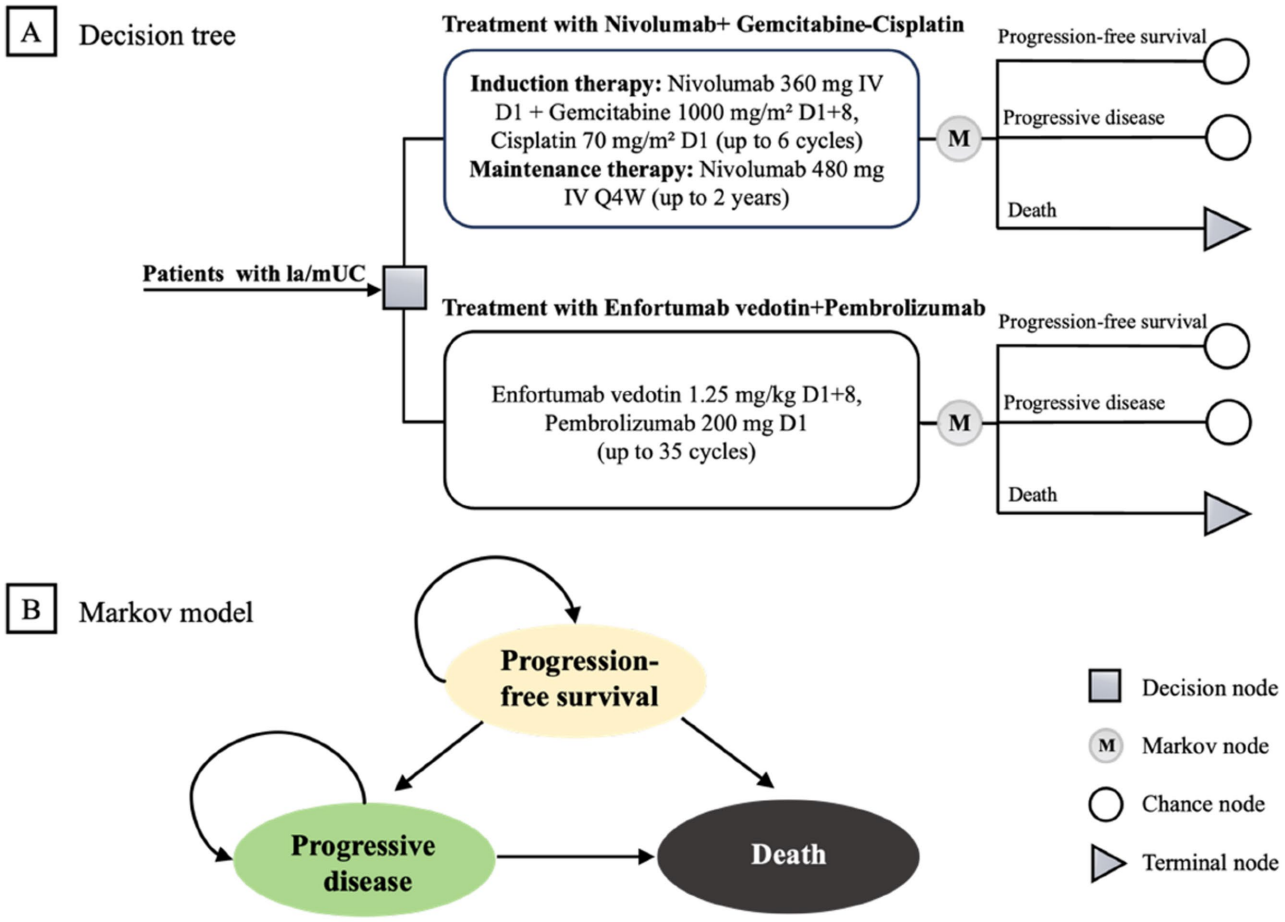
Markov model structure. **(A)** Decision tree. **(B)** Markov model. la/mUC, locally advanced or metastatic urothelial carcinoma.

We constructed a Markov model to compare EV + P with N + GC (Figure 1). During the pre-progression phase, patients received their assigned first-line regimen until radiographic progression or intolerable toxicity. After progression or prohibitive adverse events (AEs), individuals could transition to second-line therapy and best supportive care (BSC) until death.

Our simulation proceeded in 21-day cycles (aligned with trial dosing) and ran over a 50-year horizon to approximate lifetime outcomes. Country-specific discounting was applied to both costs and health effects (3% annually in the US (18); 5% in China (16)), with a half-cycle correction. Outcomes were expressed as quality-adjusted life years (QALYs) and total costs, and cost-effectiveness was summarized as incremental cost-effectiveness ratios (ICER):


$$\text{ICER}=\frac{\text{ΔCost}}{\text{ΔQALY}}\;(\begin{array}{l} \text{ΔCost}:\text{the incremental cost},\\ \text{ΔQALY}:\text{the incremental QALY} \end{array})$$

Uncertainty was examined using one-way deterministic analyses and probabilistic Monte-Carlo sensitivity analyses. Willingness-to-pay (WTP) benchmarks were set at $150,000/QALY (US) (19), and $40,451.64/QALY(China; 3 × the Chinese 2024 GDP (20, 21)). Reporting followed the 2022 CHEERS guidance.

### 2.3 Clinical data inputs

Data for this study were partially derived from two ClinicalTrials.gov registered trials: NCT04223856 (EV-302, registered February 11, 2020) and NCT03036098 (CheckMate 901, registered January 26, 2017), accessible via https://www.clinicaltrials.gov/. Because individual patient data (IPD) were not available, PFS and OS for EV-302 and CheckMate 901 were reconstructed from the published Kaplan–Meier curves (5, 7). Digitization of Kaplan–Meier survival curves (coordinates and numbers-at-risk) was performed using WebPlotDigitizer^1^, with subsequent transformation to pseudo-IPD in R (v4.5.1) via the algorithm described by Guyot et al. (22). Subsequently, parametric modeling was performed on the reconstructed IPD, incorporating six candidate distributions: exponential, Weibull, Gompertz, log-normal, log-logistic, and generalized gamma. The final model was selected based on three critical criteria: AIC and BIC values, visual congruence between model-generated results and Kaplan–Meier curves, and the clinical rationality of the model’s projected long-term performance (23). Following the distribution evaluation, the optimal extrapolation and fitting models were confirmed as: log-normal distribution for progression-free survival (PFS) of EV + P, generalized gamma distribution for PFS of N + GC, log-logistic distribution for overall survival (OS) of EV + P, and log-normal distribution for OS of N + GC (Table 1). Parameter estimates and goodness-of-fit diagnostics, along with overlays of observed and fitted curves, are provided in the Supplement (Supplementary Tables S2, S3 and Supplementary Figures S1, S2).

**Table 1:** Model inputs.

**Parameter**	**Base-case value**	**Deterministic range**	**Distribution**	**References**
Log-Normal PFS survival model of EV + P	Meanlog = 2.6709, Sdlog = 1.4649	–	–	–
Generalized Gamma PFS survival model of N + GC	Mu = 1.9794, Sigma = 1.1635, Q = –0.6043	–	–	–
Log-Logistic OS survival model of EV + P	Shape = 1.3124, Scale = 32.1285	–	–	–
Log-Normal OS survival model of N + GC	Meanlog3.1474, Sdlog = 1.2350	–	–	–
Health utility				
PFS	0.772	0.618–0.926	Beta	(39)
PD	0.698	0.558–0.838	Beta	(39)
Disutility of adverse event (G ≥ 3)				
Peripheral sensory neuropathy	0.33	0.264–0.396	Beta	(43)
Maculopapular rash	0.032	0.026–0.038	Beta	(14)
Diarrhea	0.05	0.040–0.060	Beta	(44)
Anemia	0.07	0.056–0.084	Beta	(26)
Decreased platelet count	0.05	0.040–0.060	Beta	(26)
Decreased neutrophil count	0.20	0.160–0.240	Beta	(26)
Neutropenia	0.09	0.072–0.108	Beta	(26)
Decreased white-cell count	0.20	0.160–0.240	Beta	(26)
Risk of adverse event in EV+P group (G ≥ 3), %				
Peripheral sensory neuropathy	0.041	0.033–0.049	Beta	(5)
Rash maculopapular	0.077	0.062–0.092	Beta	(5)
Diarrhea	0.039	0.031–0.047	Beta	(5)
Anemia	0.036	0.029–0.043	Beta	(5)
Neutropenia	0.052	0.042–0.062	Beta	(5)
Risk of adverse event in N + GC group (G ≥ 3), %				
Decreased platelet count	0.076	0.061–0.0912	Beta	(7)
Neutropenia	0.188	0.150–0.2256	Beta	(7)
Decreased neutrophil count	0.145	0.116–0.174	Beta	(7)
Anemia	0.22	0.176–0.264	Beta	(7)
Decreased white-cell count	0.099	0.079–0.1188	Beta	(7)

PFS, Progression-Free Survival; OS, Overall Survival, EV+P, Enfortumab Vedotin plus Pembrolizumab; N + GC, Nivolumab plus Gemcitabine-Cisplatin.

### 2.4 Cost estimates and utility inputs

Framed within the Chinese healthcare payer perspective, the economic evaluation exclusively accounted for direct medical costs, including tumor imaging fees, administration, management of adverse events (AEs), routine monitoring, BSC and terminal care expenses (Table 2).

**Table 2:** Model cost and utility inputs.

Parameters	Value(range) for the US	Reference	Value(range) for China	Reference	Distribution
Cost ($)					
Pharmacotherapy costs, $					
Enfortumab Vedotin (20 mg)	2751.73(2201.38–3302.08)	(25)	740.14(592.11–888.17)	Local assessment	Gamma
Pembrolizumab(100 mg)	5,884.02(4707.22–7060.82)	(25)	2541.56(2033.25–3049.87)	(24)	Gamma
Nivolumab(40 mg)	1,301.04(1040.83–1561.25)	(25)	650.60(520.48–780.72)	(24)	Gamma
Gemcitabine(1,000 mg)	20.93(16.74–25.12)	(25)	5.74(4.59–6.89)	(24)	Gamma
Cisplatin(50 mg)	10.55(8.44–12.66)	(25)	8.19(6.55–9.83)	(24)	Gamma
Cost of second-line therapy per cycle					
Second-line therapy	1,358.58(1,086.86-1,630.30)	(10)	157.38(125.90–188.86)	(6)	Gamma
Cost of adverse event (per event)					
Peripheral sensory neuropathy	24,918.40(19,934.72-29,902.08)	(27)	16,089.48(12,871.58-19,307.38)	(30)	Gamma
Maculopapular rash	16,337.36(13,069.89-19,604.83)	(14)	85.81(68.65–102.97)	(30)	Gamma
Diarrhea	7,968.48(6,374.78-9,562.18)	(28)	15,016.71(12,013.37-18,020.05)	(30)	Gamma
Decreased neutrophil count	37,550.24(30,040.19-45,060.29)	(14)	116.51(93.21–139.81)	(26)	Gamma
Neutropenia	17,868.24(14,294.59-21,441.89)	(14)	116.51(93.21–139.81)	(26)	Gamma
Decreased platelet count	8,608.13(6,886.50-10,329.76)	(29)	1,525.50(1,220.40-1,830.60)	(26)	Gamma
Anemia	4,823.52(3,858.82-5,788.22)	(14)	140.55(112.44–168.66)	(26)	Gamma
Decreased white-cell count	13,506.01(10,804.81-16,207.21)	(29)	114.81(91.85–137.77)	(26)	Gamma
Other cost, $					
Best supportive care per cycle	1,374.00(1,099.20-1,648.80)	(31)	711.00(568.80–853.20)	(31)	Gamma
Tumor imaging per cycle	942.00(753.60–1,130.40)	(13)	143.00(114.40–171.60)	(13)	Gamma
Intravenous drug administration	894.00(715.200–1072.800)	(32)	404.79(323.832–485.748)	(32)	Gamma
Laboratory tests and radiological examinations	2458.04(1966.432–2949.648)	(33)	672.02(537.616–806.424)	(33)	Gamma
Hospitalization and daily care	1522.56(1218.048–1827.072)	(33)	47.32(37.856–56.784)	(33)	Gamma
Routine follow-up	318.16(254.528–381.792)	(33)	27.37(21.896–32.844)	(33)	Gamma
Terminal care	6,246.00(4,996.80-7,495.20)	(13)	1,761.00(1,408.80-2,113.20)	(13)	Gamma
Other					
Weight, kg	70.00(56.00–84.00)	(13)	65.00(52.00–78.00)	(13)	Uniform
Body surface area, m^2^	1.86(1.49–2.23)	(17)	1.72(1.38–2.06)	(16)	Uniform
Discount rate	0.03(0–0.05)	(18)	0.05(0–0.08)	(16)	Uniform

In China, unit prices for medicines were sourced as follows: enfortumab vedotin was based on the average retail price in Shenzhen and Hong Kong, China; pembrolizumab, nivolumab, gemcitabine, and cisplatin from Yaozhi (national tender prices, 2025) (24). For the US, drug prices were obtained from Drugs.com (25). Other direct medical costs, including tumor imaging fees per cycle, intravenous drug administration costs, adverse event (AE) management fees, routine monitoring expenses (encompassing laboratory tests and radiological examinations), BSC per cycle, hospitalization and daily care costs, routine follow-up fees, and terminal care expenses, were sourced from prior published studies (13, 14, 26–33). For AE management, we included only the five most frequent grade ≥ 3 events reported in the relevant trial arms in the EV-302 and CheckMate-901 (Table 1). When costs were drawn from sources published before 2025, they were inflated to 2025 values using the healthcare CPI for China (34) and the medical care CPI for the US (35). All monetary inputs are expressed in 2025 US dollars using an exchange rate of $1 = ¥7.105 (36).

Health-state utilities quantify health-related quality of life on a 0–1 scale (0 = death, 1 = full health) and were applied to survival to generate discounted QALYs (37). Building on Cheung et al.’s identification of well-validated utility assessment instruments for urothelial carcinoma populations (38), we adopted the utility values derived from registry-based EQ-5D mappings of the JAVELIN Bladder 100 trial by Kapetanakis et al. (39). Specifically, PFS and PD states were assigned values of 0.772 and 0.698, respectively (Table 1). Adverse event disutility was taken from published sources (14, 26, 28–30) and implemented as one-cycle decrements in the cycle of treatment (Table 1).

### 2.5 Sensitivity analysis

Model uncertainty was explored using both deterministic (one-way) and probabilistic approaches. In the one-way analysis, all base-case inputs were perturbed by±20% except for the risk of adverse events, which was adjusted by±10%. Discount rates followed guideline ranges: 0–5% for China and 0–8% for the US. Sensitivity analysis results are presented via tornado plots, which depict how parameter variations impact the incremental cost-effectiveness ratio (ICER). Bar lengths reflect ICER sensitivity to each parameter. Cost-related parameters were assigned Gamma distributions, discount rates were modeled using Uniform distributions, and probabilities alongside utility values were fitted with Beta distributions. Furthermore, 10,000 Monte Carlo simulations were performed to extract relevant model-derived data, enabling generation of cost-effectiveness curves and scatter plots.

Internal validity was assessed by comparing model-simulated PFS/OS (and response metrics) with the corresponding trial outcomes to confirm close alignment (Table 3 and Supplementary Table S4).

**Table 3:** Clinical data of treatment modalities and simulated data.

Treatment	1-year PFS (%)	1-year OS (%)	Reference	Model-simulated 1-year PFS (%)	Model-simulated 1-year OS (%)
N + GC	34.2	70.2	(7)	34.1	71.1
EV + P	51.4	77.7	(5)	50.8	78.1

PFS, Progression-Free Survival; OS, Overall Survival; EV+P, Enfortumab Vedotin plus Pembrolizumab; N + GC, Nivolumab plus Gemcitabine-Cisplatin.

## 3 Results

### 3.1 Base-case result

In the US, EV + P was associated with total costs of $1,863,624.32 and 3.34 QALYs, while N + GC incurred costs of $881,979.07 and 2.36 QALYs. Incremental analysis of EV + P versus N + GC demonstrated incremental costs of $981,645.25 and incremental QALYs of 0.98, resulting in an ICER of $1,001,626.19/QALY. This value substantially exceeds the US WTP threshold of $150,000/QALY, indicating EV + P is not cost-effective relative to N + GC in the US context. In China, EV + P generated total costs of $485,374.69 and 2.95 QALYs, compared with $203,811.61 and 2.15 QALYs for N + GC. The incremental cost for EV + P versus N + GC was $281,563.08 with incremental QALYs of 0.80, yielding an ICER of $351,960.68/QALY. This ICER remains well above the Chinese WTP benchmark of $40,451.64/QALY, confirming EV + P is also not cost-effective in the Chinese setting (Table 4).

**Table 4:** Summary of 50-year simulation cost and health outcome results in the base-case analysis.

Treatment	Cost	Inc. costs	QALYs	Inc. QALYs	ICER($/QALY)
US					
N + GC	881,979.07	–	2.36	–	–
EV + P	1,863,624.32	981,645.25	3.34	0.98	1,001,626.19
China					
N + GC	203,811.61	–	2.15	–	–
EV + P	485,374.69	281,563.08	2.95	0.80	351,960.68

Inc., incremental; QALYs, Quality-Adjusted Life Years; ICER, Incremental Cost-Effectiveness Ratio; N + GC, Nivolumab plus Gemcitabine-Cisplatin; EV+P, Enfortumab Vedotin plus Pembrolizumab.

Curves show the proportion of the cohort in each state by cycle, allowing visual comparison of disease control and mortality dynamics (Supplementary Figure S3). Over the 50-year model horizon with 21-day cycles, by the end of the final cycle, 99.6% of patients in the N + GC group had died, while 97.9% of patients in the EV + P group had died.

### 3.2 Sensitivity analysis

**Figure 2:**
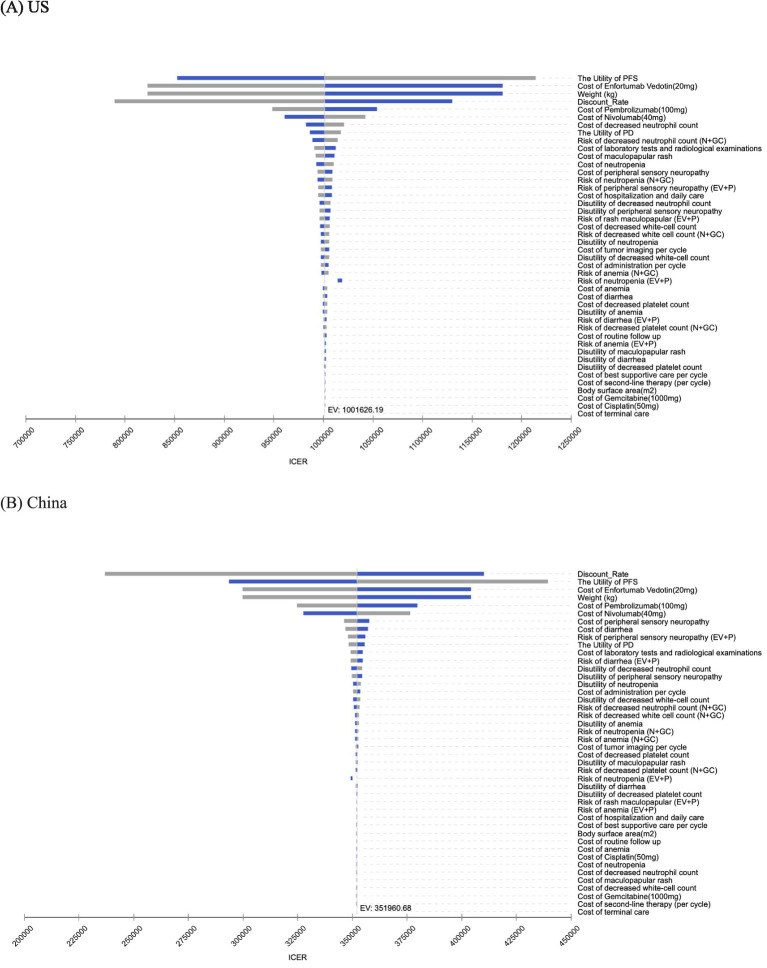
One-way deterministic sensitivity analysis for EV + P vs. N + GC. **(A)** US. **(B)** China. EV + P, Enfortumab Vedotin plus Pembrolizumab; N + GC, Nivolumab plus Gemcitabine-Cisplatin.

The primary drivers of the ICER were broadly consistent across different settings. In the US model, the five most influential parameters were: the utility of PFS, unit price of enfortumab vedotin, assumed US body weight, discount rate in the US, and unit price of pembrolizumab. In the China model, the top five parameters were: discount rate in China, the utility of PFS, unit price of enfortumab vedotin, assumed China body weight, and unit price of pembrolizumab. Other inputs exerted only minor effects in the tornado plots (Figure 2).

**Figure 3:**
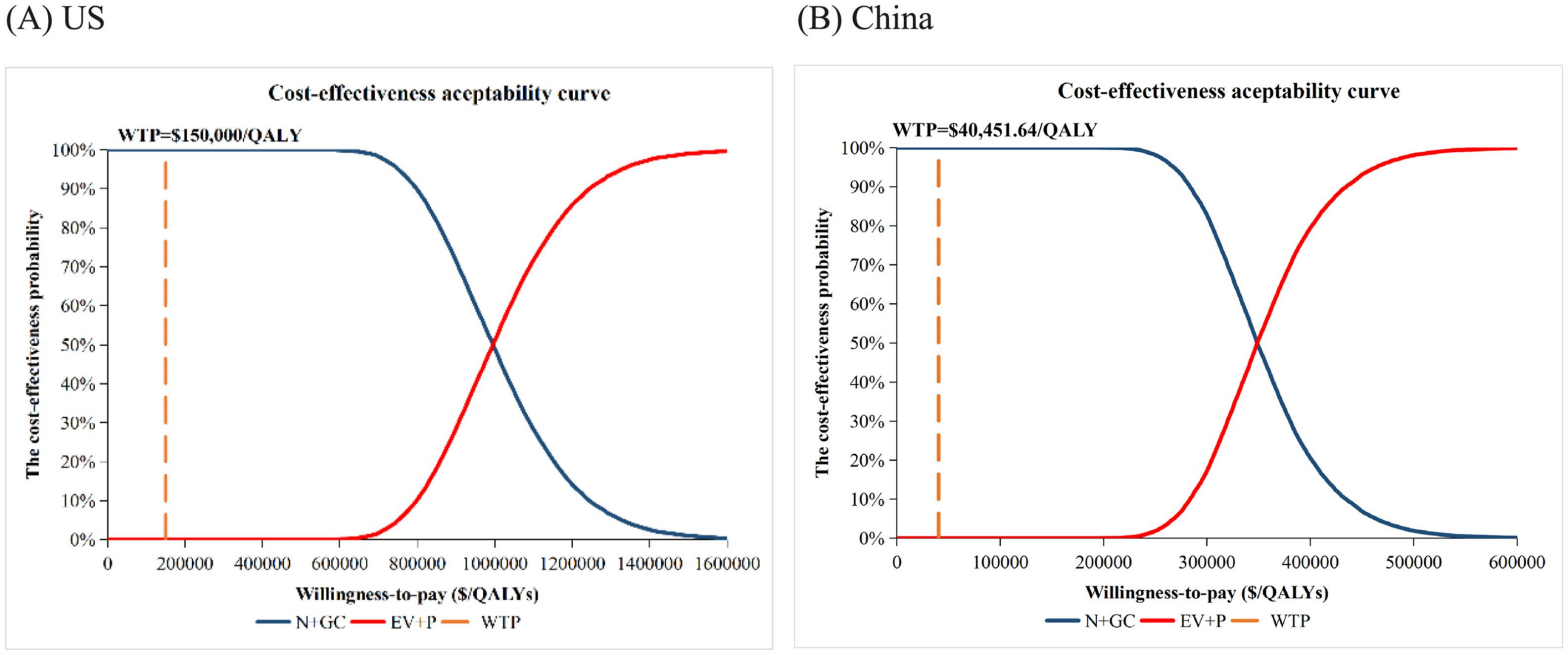
Cost-effectiveness acceptability curves. **(A)** US. **(B)** China. WTP, Willingness-to-pay; EV + P, Enfortumab Vedotin plus Pembrolizumab; N + GC, Nivolumab plus Gemcitabine-Cisplatin.

All dots represent one of 10,000 Monte Carlo draws from the probabilistic sensitivity analysis (PSA), with the ellipse enclosing roughly the 95% confidence region. All dots lie below the country-specific willingness-to-pay (WTP) thresholds of $40,451.64/QALY for China and $150,000/QALY for the US, indicating a higher probability that N + GC is the cost-effective option (Supplementary Figure S4). The cost-effectiveness acceptability curves showed a 0% probability that EV + P is cost-effective at $150,000/QALY (US) or $40,451.64/QALY (China) (Figure 3). Taken together, the deterministic and probabilistic findings confirm that N + GC remains the economically preferred first-line option under both US and Chinese payment thresholds.

### 3.3 One-way price scenario

Although enfortumab vedotin (EV) has been launched in mainland China, no unified national tender/negotiated price was publicly available at the time of model construction. We therefore adopted the average market price in Shenzhen and Hong Kong as the baseline and converted it to 2025 US dollars. To address the resulting pricing uncertainty, we prespecified wide price scenarios and threshold analyses; given weight-based dosing, the per-cycle EV acquisition cost was treated as the key parameter and varied extensively in one-way sensitivity testing to cover potential post-negotiation levels.

The one-way price analysis demonstrated that in the US, EV + P would achieve cost-effectiveness only if its price were reduced by at least 95.05%, bringing the unit price of Enfortumab Vedotin (20 mg) to $136.16. In contrast, even if the unit cost of EV were reduced to $0 in China, EV + P would still not be cost-effective at the WTP threshold specific to China of $40,451.64/QALY (Supplementary Figure S5). This finding indicates that price cuts on EV alone are unlikely to overturn the economic disadvantage of EV + P versus N + GC.

### 3.4 Two-way price scenario

To identify the price ranges at which EV + P could become cost-effective versus N + GC, we performed a two-way price analysis jointly varying the acquisition costs of enfortumab vedotin plus pembrolizumab. In the China model, a combined price reduction of 80% was required to bring the ICER below the WTP threshold of $40,451.64/QALY; in the US model, a combined reduction of 75% was needed to lower the ICER below $150,000/QALY (Supplementary Figure S6 and Table 5).

**Table 5:** Price sensitivity analysis of combined price reductions for EV + P vs. N + GC in the US and China.

Cost of EV+P	Inc. cost ($)	Inc. QALYs	ICER ($/QALY)	WTP ($/QALY)	Comment
US					
Baseline	981,645.25	0.98	1,001,626.19	150,000.00	Not cost-effective
45%	356,296.18	0.98	363,548.43		Not cost-effective
35%	242,596.35	0.98	247534.29		Not cost-effective
25%	128,896.52	0.98	131,520.15		Cost-effective
20%	72,046.60	0.98	73,513.08		Cost-effective
China					
Baseline	281,563.08	0.80	351,960.68	40,451.64	Not cost-effective
45%	106,374.90	0.80	132,971.21		Not cost-effective
35%	74,522.51	0.80	93,154.94		Not cost-effective
25%	42,670.11	0.80	53,338.67		Not cost-effective
20%	26,743.91	0.80	33,430.54		Cost-effective

EV + P, Enfortumab Vedotin plus Pembrolizumab; N + GC, Nivolumab plus Gemcitabine-Cisplatin; Inc., incremental; WTP, Willingness-to-pay; QALY, Quality-Adjusted Life Year; ICER, Incremental Cost-Effectiveness Ratio.

## 4 Discussion

Over the past decades, first-line therapy for mUC was anchored in platinum-based chemotherapy. In EV-302, EV + P nearly doubled median PFS (12.5 vs. 6.3 months) and prolonged median OS to 33.8 months versus 15.9 months with chemotherapy, with hazard ratios ~0.45–0.51 across primary and updated readouts (5). In CheckMate-901, N + GC improved OS (median 21.7 vs. 18.9 months; HR 0.78) and PFS (HR 0.72), with 12-month PFS rates 34.2% vs. 21.8% over chemotherapy (7). Yet, contemporary pharmacoeconomic studies from both China and the US have repeatedly found that these innovative regimens are often not cost-effective compared with gemcitabine-cisplatin, primarily because of high drug acquisition costs. Despite the clear clinical benefits of EV + P, its economic value remains uncertain. Consequently, a cost-effectiveness analysis of EV + P versus N + GC in the Chinese and US healthcare contexts is highly relevant, especially for patients and healthcare decision-makers who prioritize therapeutic efficacy while being mindful of cost affordability. Accordingly, we conducted a comparative economic analysis of these two evidence-based first-line strategies.

In both the Chinese and US healthcare settings, first-line EV + P provided improved health outcomes compared with N + GC but at substantially higher costs, resulting in ICERs well above the respective country-specific WTP thresholds. Consequently, EV + P is not cost-effective relative to N + GC in either setting under current pricing and reimbursement structures, with N + GC remaining the economically preferred option. Deterministic sensitivity analyses revealed broadly consistent patterns in both countries: ICER variation was dominated by the discount rate, utility of PFS, unit price of enfortumab vedotin, assumed body weight (reflecting weight-based dosing), and unit price of pembrolizumab, while other inputs exerted only minor influence. Probabilistic sensitivity analysis results and cost-effectiveness acceptability curves indicated that EV + P is not cost-effective at the US or Chinese WTP thresholds, confirming the base-case conclusion is robust to wide parameter uncertainty.

This study conducted one-way and two-way price sensitivity analyses for the EV + P regimen to address the absence of a unified national negotiated or procurement price for enfortumab vedotin in mainland China during the model development phase. The baseline analysis used the average market price from Shenzhen and Hong Kong, converted to 2025 US dollars, with predefined broad price scenarios and threshold analyses to mitigate pricing uncertainty. The one-way sensitivity analysis treated the unit price of enfortumab vedotin as the key parameter. Results indicated that in the US, the price of enfortumab vedotin would need to be reduced by at least 95.05%, lowering the 20 mg unit price to $136.16 for the EV + P regimen to potentially achieve cost-effectiveness. In China, however, even if the unit cost of enfortumab vedotin were reduced to $0, the ICER of the EV + P regimen would still exceed WTP threshold. This suggests that reducing the price of enfortumab vedotin alone cannot reverse the economic disadvantage of the EV + P regimen compared to the N + GC regimen. The model allows for the future direct substitution of officially announced prices in mainland China, enabling conclusions to be updated without model reconstruction, ensuring practical applicability in clinical decision-making. The two-way sensitivity analysis, which simultaneously adjusted the unit prices of enfortumab vedotin plus pembrolizumab, revealed that a combined price reduction of 80% for both drugs is required in China, and 75% in the US, for the ICER of the EV + P regimen to fall below the respective national WTP thresholds.

From a policy-making perspective, a key finding of this study is that isolated drug price reductions are insufficient for the EV + P regimen to become a cost-effective treatment option. Two-way sensitivity analysis quantifies this challenge: within the healthcare payment systems of the US and China, the regimen requires combined price reductions of approximately 75 and 80%, respectively, for enfortumab vedotin plus pembrolizumab for its incremental cost-effectiveness ratio to reach each country’s WTP threshold. Neither EV nor P has undergone negotiations for inclusion in China’s National Reimbursement Drug List (NRDL). This result underscores the absolute necessity for coordinated pricing negotiations, with the NRDL negotiation mechanism emerging as a core policy tool to achieve such substantial price reductions. Existing research inferences (40) have indicated that NRDL-driven price negotiations for the EV + P regimen could align drug prices with China’s national WTP threshold, thereby significantly enhancing its cost-effectiveness in China. Given the stark differences in healthcare systems across countries, differentiated feasible pathways must be designed for China and the US. In China, beyond leveraging NRDL negotiations and volume-based procurement policies, practical cost-containment strategies validated in oncology settings should also be incorporated—such as vial sharing (to reduce unit-dose wastage and lower actual costs for patients and payers) and the potential launch of biosimilars (40). Both measures serve as critical complementary approaches to NRDL pricing in improving the economic value of EV + P. By bundling EV and pembrolizumab for “package pricing” negotiations, with NRDL inclusion and guaranteed long-term purchase volumes as leverage, the marginal costs of pharmaceutical companies can be effectively reduced, creating room for significant price cuts. However, considering the inherently high research and development costs of EV as an innovative antibody-drug conjugate (ADC), mandating an 80% price reduction in the short term may deter the introduction of future innovative drugs. Therefore, a more pragmatic “phased price reduction” strategy is feasible: initially, a combined price reduction target of 30–40% could be set, allowing EV + P to be prioritized for high-risk subgroups of patients with extremely poor prognoses and the greatest expected benefits. In the long run, as patents expire, competition from biosimilars intensifies, and real-world efficacy data accumulates, mechanisms such as “outcome-based stratified pricing” and “international price referencing” (40) can be leveraged to gradually approach the target price. In the US, the lack of a centralized pricing system creates greater reliance on market-based instruments like “value-based pricing.” Payers can enter into outcomes-based risk-sharing agreements with pharmaceutical companies, linking drug payment to real-world endpoints such as survival benefits, supplemented by drug specification optimization (e.g., introducing low-dose formulations to reduce waste) and reimbursement policy adjustments (e.g., inclusion in special disease insurance plans). Although achieving a 75% reduction faces greater resistance in a free-market context, by precisely aligning payment with clinical value and strictly limiting use to the most appropriate patient populations, its cost-effectiveness can be gradually optimized without exceeding budget constraints. In summary, although the EV + P regimen is not economically viable at current prices, through context-specific, phased, and multi-pronged policy interventions, there is potential to systematically steer its price towards its true clinical value, ultimately allowing its exceptional efficacy to benefit a broader patient population.

### 4.1 Limitation

This study has four limitations. First, we must acknowledge that indirect comparisons across trials may impact the results. Clinical inputs were derived from two phase III trials (EV-302 and CheckMate-901). While the key baseline characteristics of the study populations in these two trials were generally consistent (Supplementary Table S1), with each treatment regimen compared against platinum-based chemotherapy in its respective trial, this analysis relies on an unanchored indirect treatment comparison (ITC) due to the absence of direct head-to-head evidence between EV + P and N + GC. Residual heterogeneity cannot be fully eliminated without IPD or anchor population adjustment; however, since neither trial has publicly released IPD, anchor population adjustment was not feasible. This limitation is particularly noteworthy when integrating trial data into a Markov model, as Rieger et al. (12) similarly observed in their cost-effectiveness analysis of mUC treatments—they emphasized that unanchored ITCs without anchor population adjustment may introduce biases into survival parameter extrapolation and transition probability calculations, thereby affecting the reliability of long-term cost-effectiveness estimates derived from the model. The relative efficacy estimates from this ITC should therefore be interpreted as exploratory, as trial-level heterogeneity may introduce biases. Specifically, differences between the two trials in post-progression treatment patterns (e.g., access to subsequent lines of therapy), geographic distribution of participants (e.g., the proportion of Asian vs. Western patients), and follow-up intensity (e.g., the frequency of imaging assessments to detect progression) could compromise the comparability of survival outcomes. These unmeasured confounders may limit the robustness of inferences about relative effectiveness and, in turn, the reliability of the cost-effectiveness results. That said, our core finding regarding treatment efficacy alignment—namely that EV + P confers the greatest survival benefit among first-line regimens—has been validated by Gasperoni et al. (41), who conducted direct indirect comparisons of multiple mUC first-line treatments using the IPDfromKM method. Their study, which enabled precise head-to-head efficacy ranking, also confirmed that EV + P outperforms N + GC and other regimens in terms of overall survival, supporting the consistency of our efficacy-related conclusions despite the limitations of unanchored ITC. Existing studies have further shown that when patient baselines are similar, minor differences between trials are unlikely to alter the overall conclusions (42). Second, the disutility of AEs was limited to the most common grade three or higher events, which may underestimate QoL losses associated with low-grade but persistent toxicities. Third, the consistency of cost calculations and pricing. Direct medical costs were compiled from national tender/compendia and prior studies for the US and China. However, unit prices, vial-level wastage, healthcare institution location-based fees, and post-contract net prices may vary across institutions and time points. The unified price of enfortumab vedotin in mainland China was not publicly available at the time of model construction; therefore, we used the average reference price from Shenzhen and Hong Kong (valued in 2025 US dollars) and explored a wide range of one-way and two-way price scenarios. While this mitigates uncertainty, actual procurement terms (reimbursement, vial sharing, access policies) may alter the absolute incremental cost-effectiveness ratio. Fourth, our results are intended to provide references for healthcare payers and do not include indirect costs, so conclusions from a societal perspective may differ.

## 5 Conclusion

This pharmacoeconomic evaluation compared two first-line regimens for locally advanced or metastatic urothelial carcinoma. It found that despite demonstrating superior survival benefits, EV + P is not cost-effective compared to N + GC under current pricing in both China and the US. Therefore, N + GC currently represents the economically preferred first-line treatment option in both countries, findings that are most applicable to the cisplatin-eligible populations enrolled in the EV-302 and CheckMate-901 trials and should be cautiously extrapolated to other patient subgroups.

For EV + P to become a cost-effective alternative, substantial price reductions or reimbursement revisions are necessary. Additional strategies such as vial-sharing, dose optimization, and biomarker-guided patient selection could further enhance its economic value.

## Data Availability

The original contributions presented in the study are included in the article/Supplementary material, further inquiries can be directed to the corresponding author/s.

## Footnotes

1
https://automeris.io/
