# Supplementary material for: First-line enfortumab vedotin-pembrolizumab versus nivolumab plus gemcitabine-cisplatin in metastatic urothelial cancer: a cost-effectiveness study
Source: Front Public Health. 2026 Feb 12;14:1723784. doi: 10.3389/fpubh.2026.1723784 (PMC12935998; doi:10.3389/fpubh.2026.1723784)
Supplement: Supplementary file 1 [file Data_Sheet_1.pdf]

Table S 1 Clinical and Demographic Characteristics of the Patients at Baseline

**Characteristic**	**EV+P (N=442)(1)**	**N+GC (N=304)(2)**
Median age, years (range)	69 (37–87)	65 (32–86)
Sex, %		
Male	77.8	77.6
Female	22.2	22.4
Race/ethnicity, %		
White	69.7	69.4
Asian	22.4	24.7
Black	0.7	0.0
Other	7.2	5.9
ECOG performance status, %		
0	50.5	53.3
1	46.2	46.1
>1	3.4	0.7
Disease status/stage at randomization, %		
Metastatic	95.2	85.9
Locally advanced/locally unresectable–nonmetastatic	4.8	13.5
Not reported	-	0.7
Liver metastasis, %		
Yes	22.6	21.1
Geographic region		
Europe	38.9	44.1
Rest	37.8	49.7

Table S 2 The results of parametric models for Nivolumab plus Gemcitabine-cisplatin

**Parameters**	**Distribution**	**Exponential**	**Weibull**	**Gamma**	**Generalized Gamma**	**Gompertz**	**Log-Normal**	**Log-Logistic**	**Minimum**
**PFS**	AIC	1597.981	1599.532	1599.371	**1540.321**	1575.818	1547.699	1547.528	1540.321
	BIC	1601.698	1606.966	1606.805	**1551.472**	1583.252	1555.133	1554.962	1551.472
	mean/shape/mu				**1.9794**				
	sd/scale/sigma				**1.1635**				
	Q				**-0.6043**				
**OS**	AIC	1545.007	1544.629	1542.796	1534.368	1546.759	**1532.403**	1533.460	1532.403
	BIC	1548.724	1552.063	1550.230	1545.519	1554.193	**1539.837**	1540.894	1539.837
	mean/shape/mu						**3.1474**		
	sd/scale/sigma						**1.2350**		

Abbreviation: OS, overall survival; PFS, progression-free survival; AIC, akaike information criterion; BIC, bayesian information criterion

Table S 3 The results of parametric models for Enfortumab Vedotin plus Pembrolizumab

**Parameters**	**Distribution**	**Exponential**	**Weibull**	**Gamma**	**Generalized Gamma**	**Gompertz**	**Log-Normal**	**Log-Logistic**	**Minimum**
**PFS**	AIC	2200.639	2196.143	2200.162	2158.682	2165.876	**2158.392**	2164.377	2158.392
	BIC	2204.730	2204.326	2208.344	2170.956	2174.059	**2166.575**	2172.560	2166.575
	mean/shape/mu						**2.6709**		
	sd/scale/sigma						**1.4649**		
**OS**	AIC	1972.039	1969.690	1969.179	1970.273	1972.186	1974.188	**1967.858**	1967.858
	BIC	1976.056	1977.873	1977.362	1982.547	1980.369	1982.371	**1976.041**	1975.101
	mean/shape/mu							**1.3124**	
	sd/scale/sigma							**32.1285**	

Abbreviation: OS, overall survival; PFS, progression-free survival; AIC, akaike information criterion; BIC, bayesian information criterion

Table S 4 The median survival time of original and reconstructed Kaplan Meier survival curve.

Kaplan Meier survival curve	mOS (95% CI)	mPFS (95% CI)
N+GC: Original	21.7 (18.6-26.4)	7.9 (7.6-9.5)
**N+GC: Reconstructed**	21.7 (18.9-27.1)	7.92 (7.65-9.6)
EV+P: Original	33.8 (26.1-39.3)	12.5 (10.4-16.6)
**EV+P: Reconstructed**	33.7 (26.0-NA)	12.56 (10.41-16.5)

Abbreviation: EV+P, Enfortumab Vedotin plus Pembrolizumab; N+GC, Nivolumab plus Gemcitabine-Cisplatin; mPFS, median progression-free survival; mOS, median overall survival.

(A)

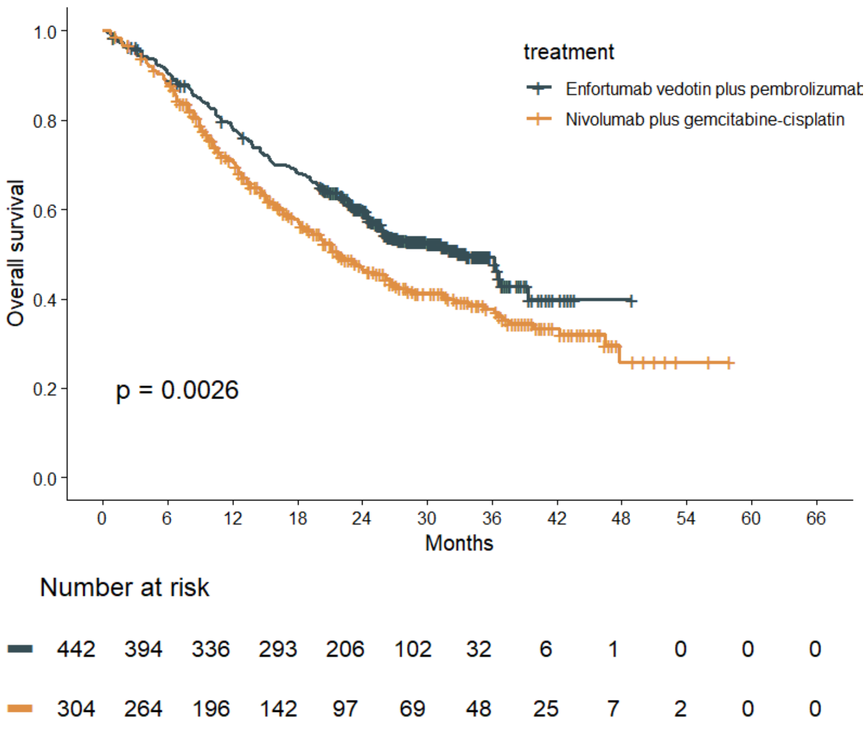

(B)

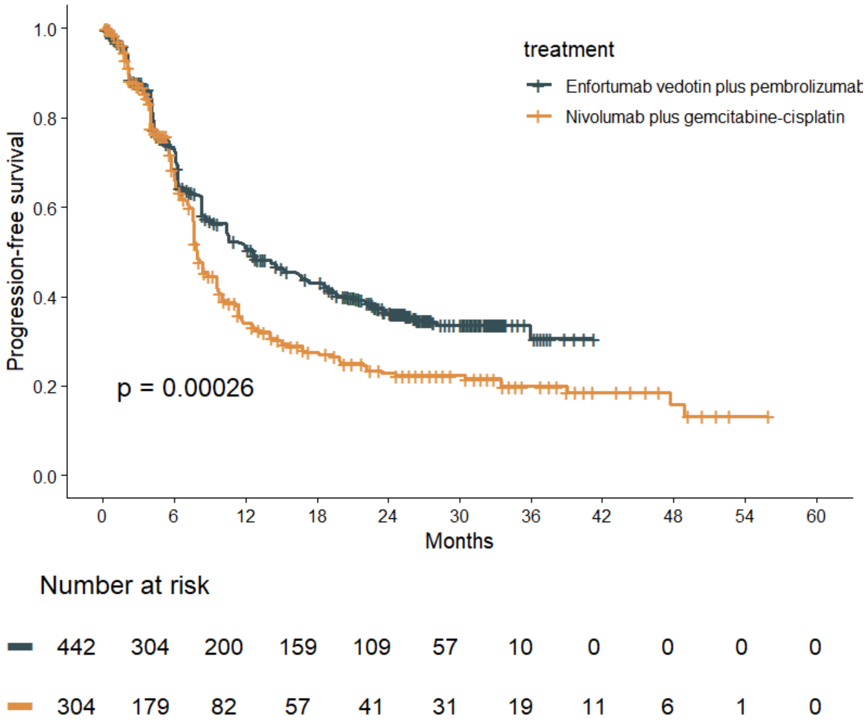

Figure S 1 Reconstruction of Kaplan Meier survival curve. (A) Overall survival curve. (B) Progression-free survival curve.

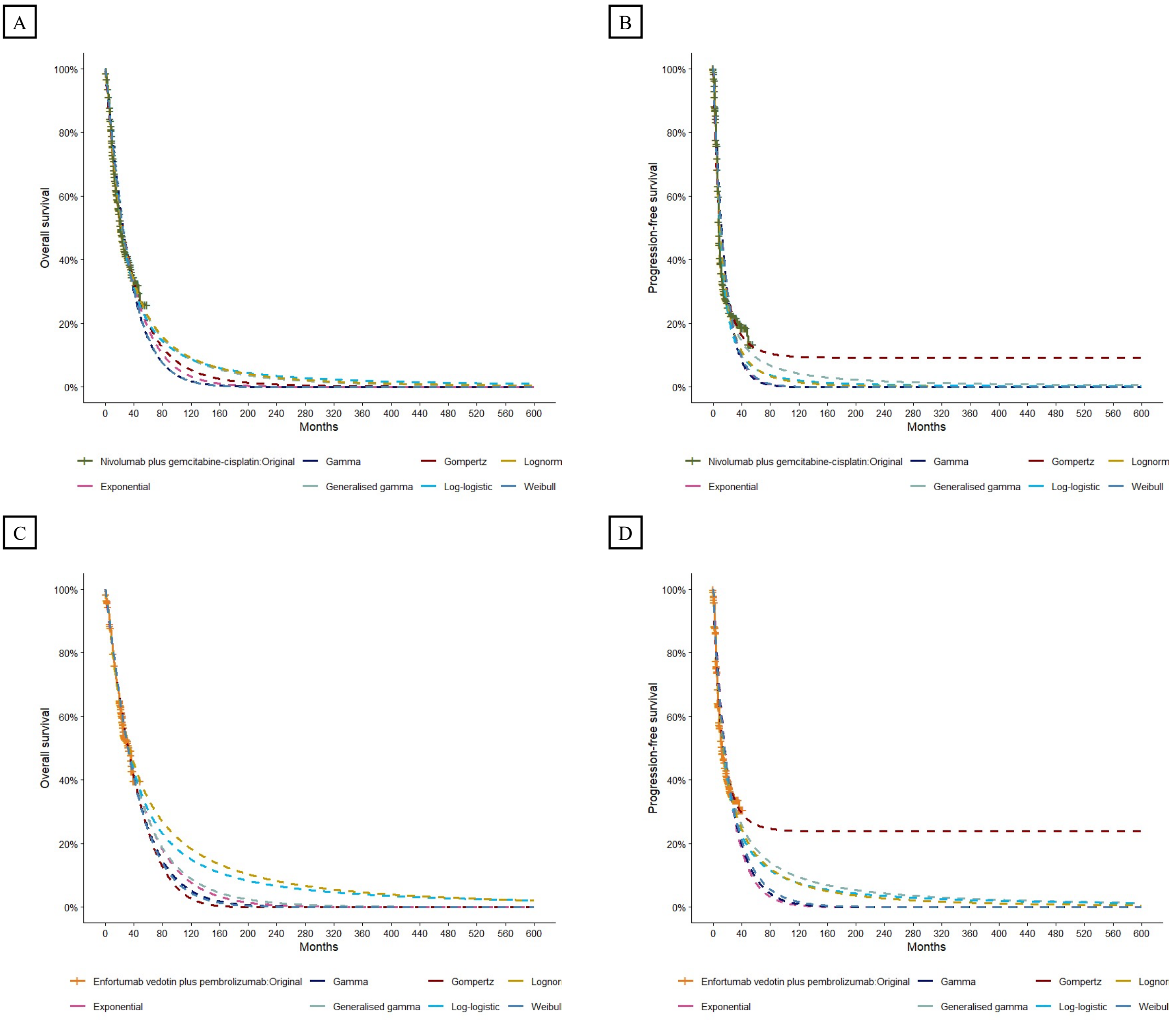

Figure S 2 Fitting and extrapolation of Kaplan Meier survival curve. (A)The results of N+GC OS curve. (B)The results of N+GC PFS curve. (C)The results of EV+P OS curve. (D)The results of EV+P PFS curve.

(A) Nivolumab plus Gemcitabine-Cisplatin

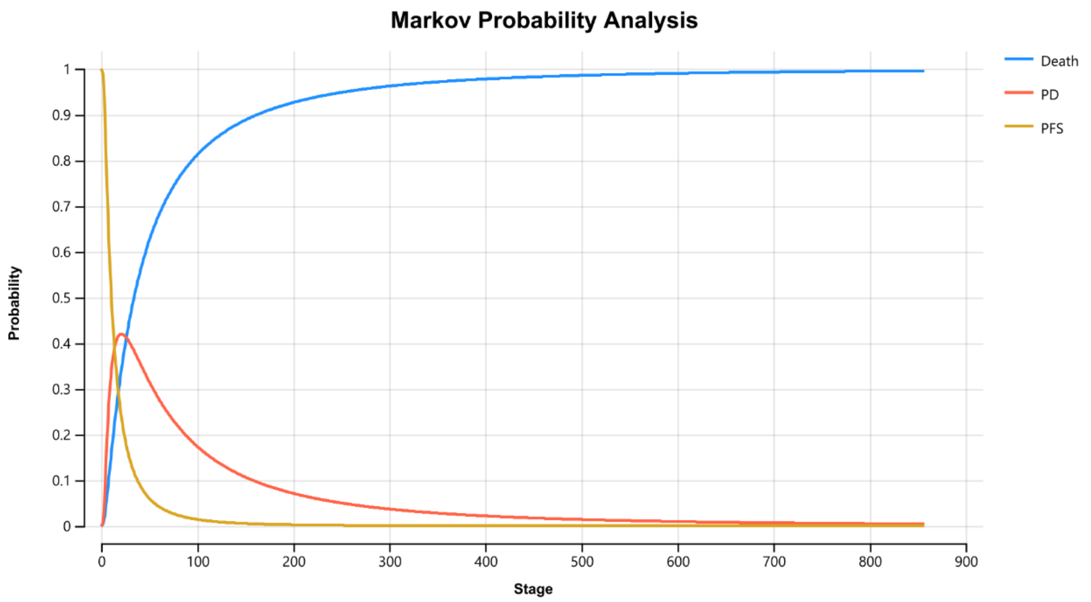

(B) Enfortumab Vedotin and Pembrolizumab

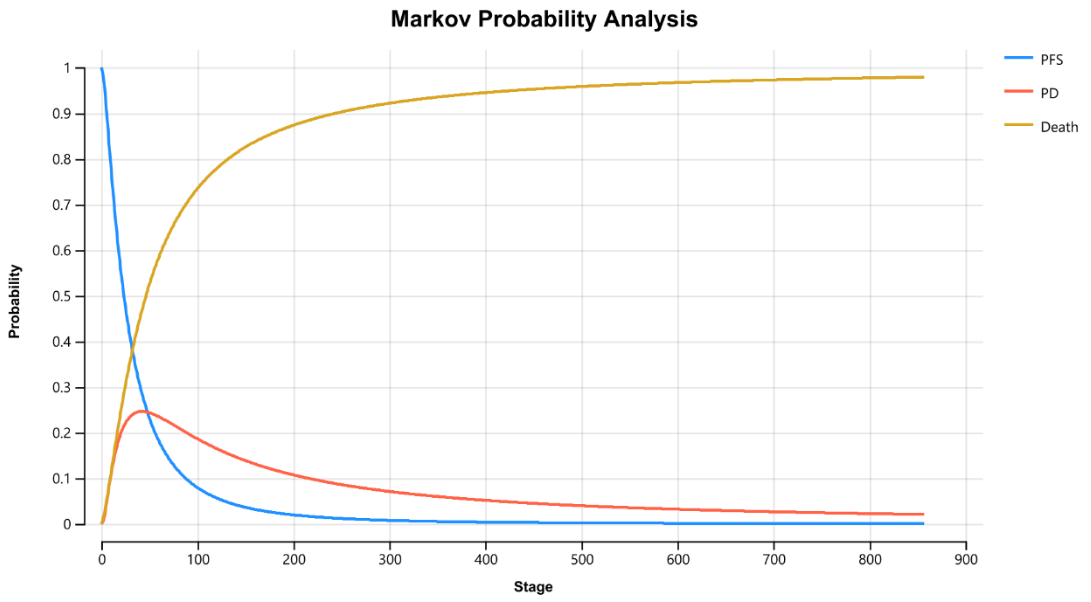

Figure S 3 Population distributions in different states. (A) Nivolumab plus Gemcitabine-Cisplatin, (B) Enfortumab Vedotin plus Pembrolizumab

(A) US

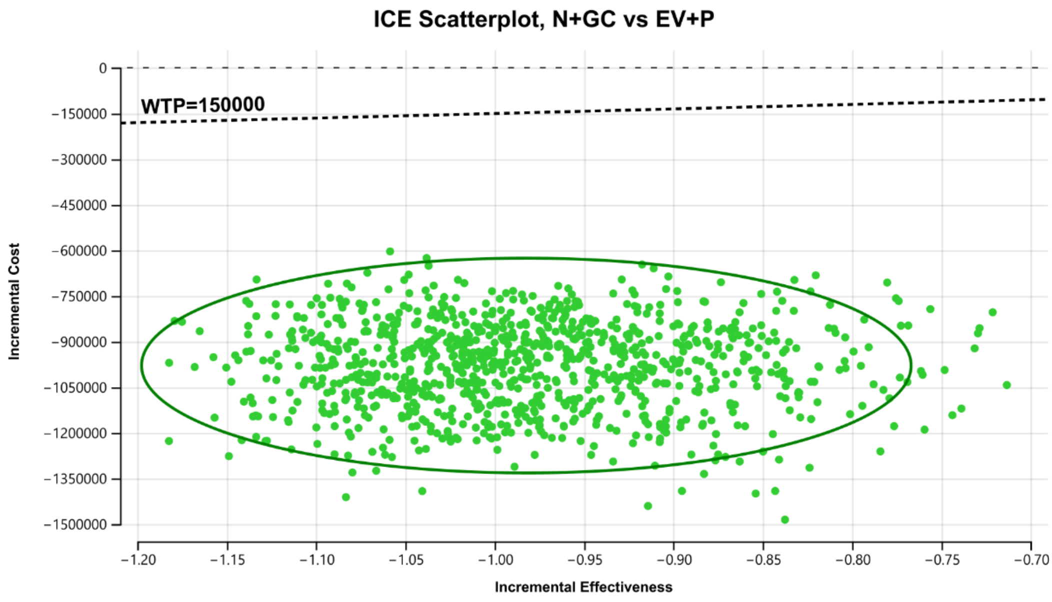

(B) China

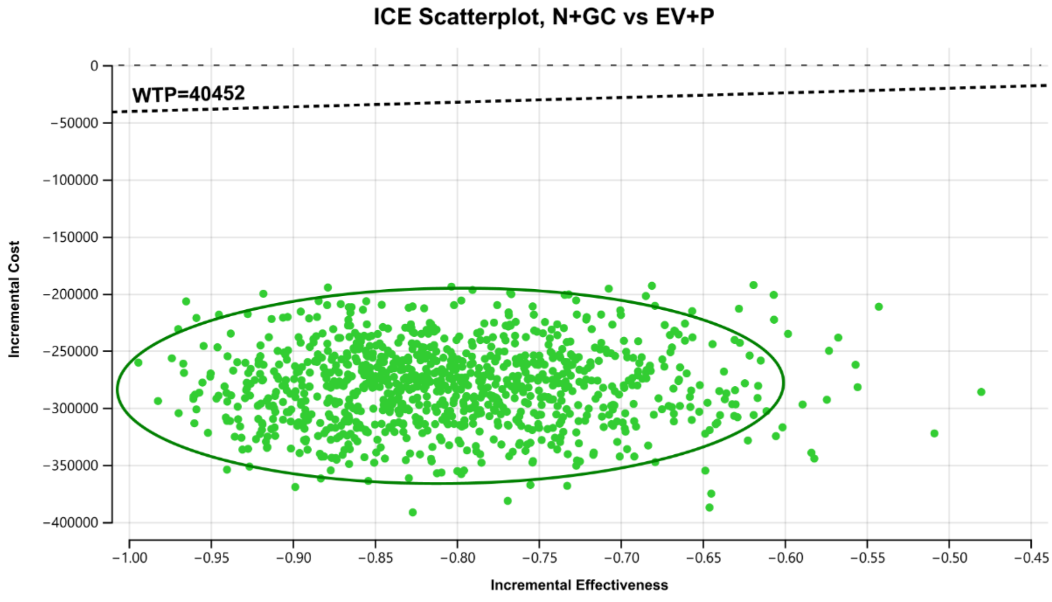

Figure S 4 Incremental Cost-Effectiveness (ICE) Scatterplots.

(A) US

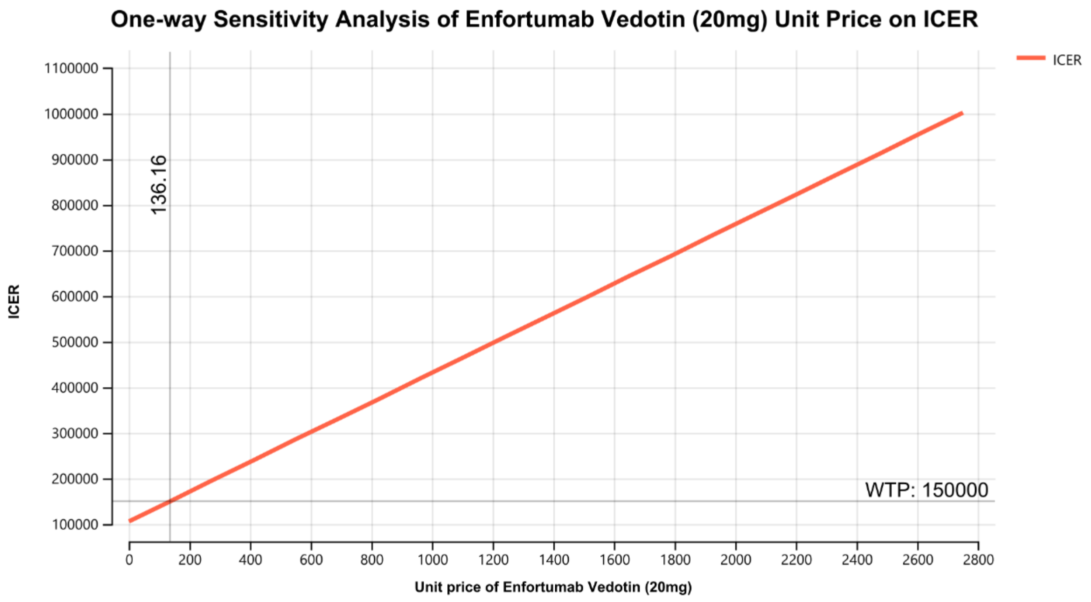

(B) China

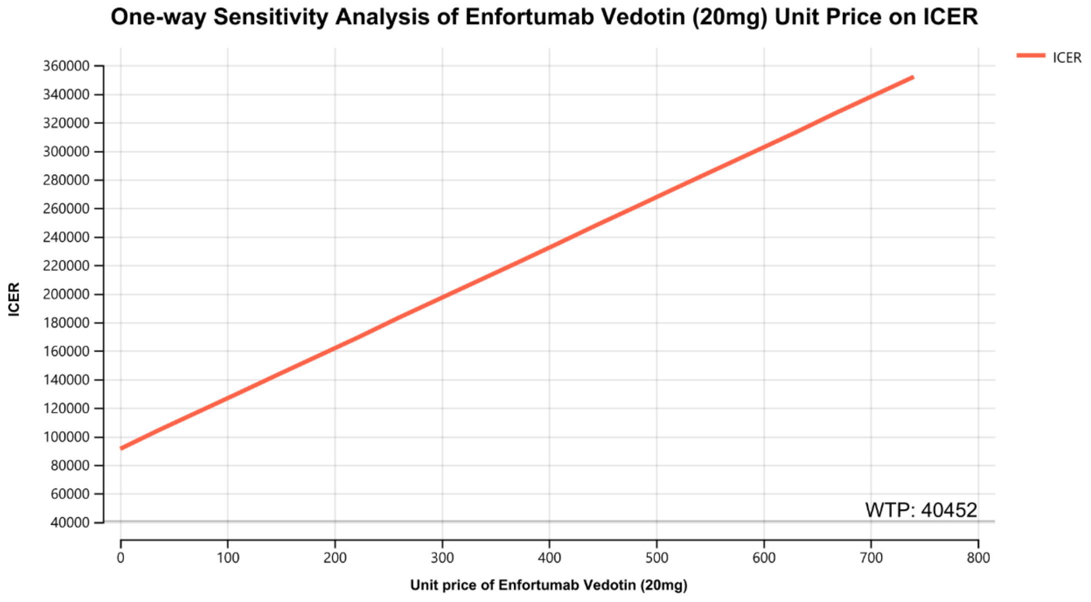

Figure S 5 One-way Sensitivity Analysis of Enfortumab Vedotin (20mg) Unit Price on ICER

(A) US

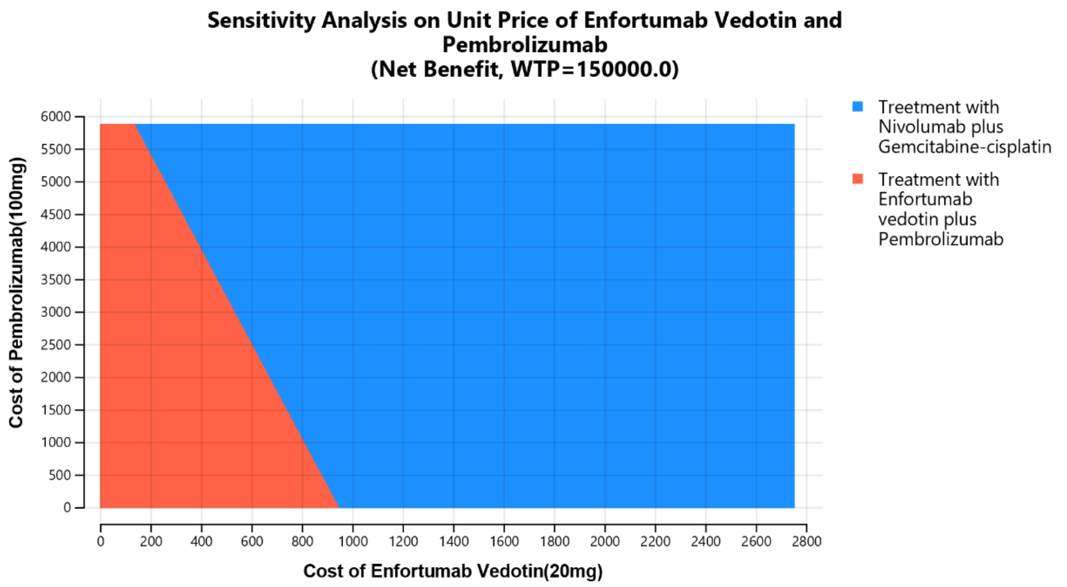

(B) China

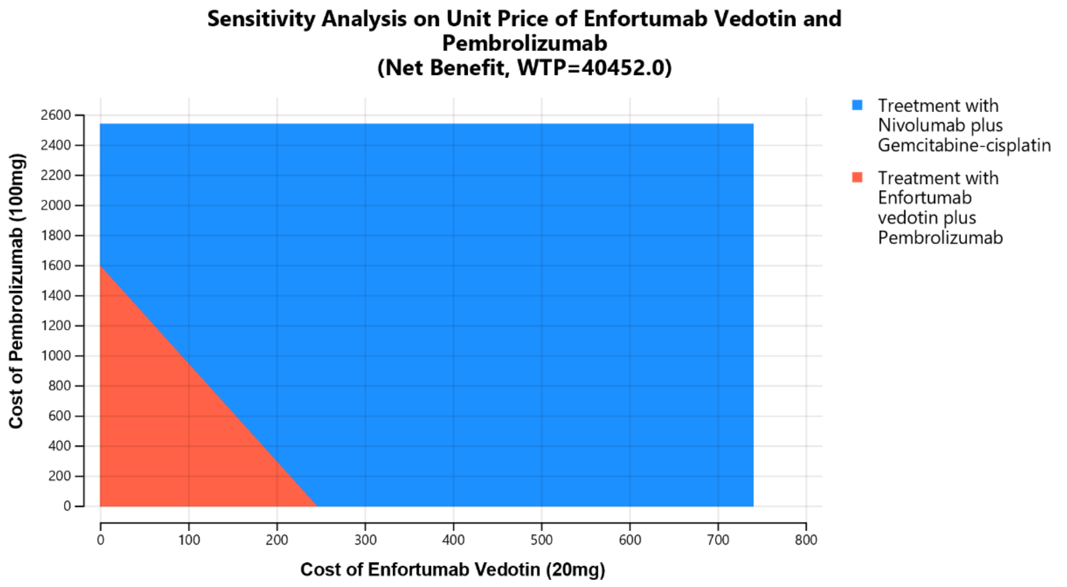

Figure S 6 Two-way Sensitivity Analysis of Enfortumab Vedotin (20mg) and Pembrolizumab (100mg) Unit Prices on Net Benefit

1. Powles T, Valderrama BP, Gupta S, Bedke J, Kikuchi E, Hoffman-Censits J, et al. Enfortumab Vedotin and Pembrolizumab in Untreated Advanced Urothelial Cancer. N Engl J Med. 2024;390(10):875-88.
2. van der Heijden MS, Sonpavde G, Powles T, Necchi A, Burotto M, Schenker M, et al. Nivolumab plus Gemcitabine-Cisplatin in Advanced Urothelial Carcinoma. N Engl J

Med. 2023;389(19):1778-89.
